# A Tight-Knit Family: The Medium-Chain Dehydrogenase/Reductases of Monoterpene Indole Alkaloid Biosynthesis

**DOI:** 10.1021/acs.biochem.5c00234

**Published:** 2025-06-19

**Authors:** Samuel C. Carr, Sarah E. O’Connor

**Affiliations:** Department of Natural Product Biosynthesis, 28298 Max Planck Institute for Chemical Ecology, 07745 Jena, Germany

**Keywords:** medium-chain dehydrogenase/reductase, alcohol dehydrogenase, plant natural products, monoterpene indole alkaloid, iminium reduction, carbonyl reduction, noncanonical activity, catalytic architecture

## Abstract

Medium-chain dehydrogenases/reductases (MDRs) are enzymes that are well-known for catalyzing the reversible reduction of ketones or aldehydes or oxidation of alcohols. However, the biosynthetic pathways of the monoterpene indole alkaloids (MIAs), an important class of natural products derived from plants, highlight that MDRs can also catalyze 1,2- and 1,4-α,β-unsaturated iminium reductions, as well as 1,4-α,β-unsaturated carbonyl reduction. The noncanonical activities of these MDRs correlate with distinct catalytic architectures centered on amino acid substitutions that impact catalytic zinc coordination, acid/base catalysis, and proton relay. These noncanonical MDR catalytic architectures likely arose within the MDR subfamily of cinnamyl alcohol dehydrogenases (CADs). This review summarizes the currently characterized MIA biosynthetic MDRs along with an analysis of the catalytic mechanisms, structural underpinnings, and phylogeny.

## Graphical abstract

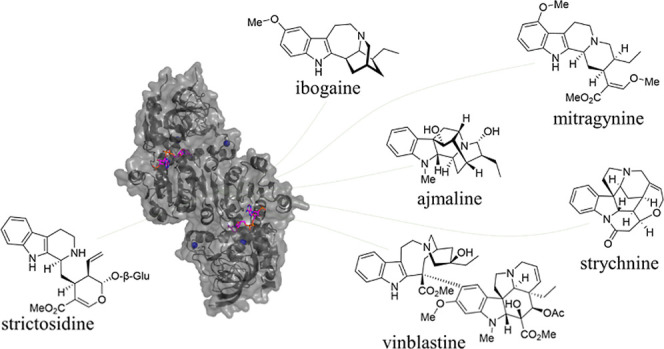

## Introduction

Monoterpene indole alkaloids (MIAs) comprise a large and diverse family of plant specialized metabolites with over 2500 known compounds, biosynthesized primarily by members of the Gentianales order (, and families).^1^ The MIA family harbors a wide range of biologically active compounds including vincristine/vinblastine (anticancer, ),^2–4^ quinine (antimalaria, spp.),^5^ mitragynine (Kratom opiate, spp.),^6,7^ alstonine (antipsychotic, spp.),^8,9^ ajmalicine (antihypertensive, spp. and ),^10–12^ yohimbine (stimulant, ),^13^ ajmaline (antiarrhythmic, spp.),^14^ strychnine (pesticide, ),^15^ and ibogaine (antiaddiction, ).^16,17^ The wide range of bioactivities of MIAs stem from the diverse chemical structures observed in this class of alkaloids (Figure 1). Additional chemical diversity can be achieved through dimerization of these monomers to form bisindole MIAs, such as vincristine and vinblastine in .

**Figure 1:**
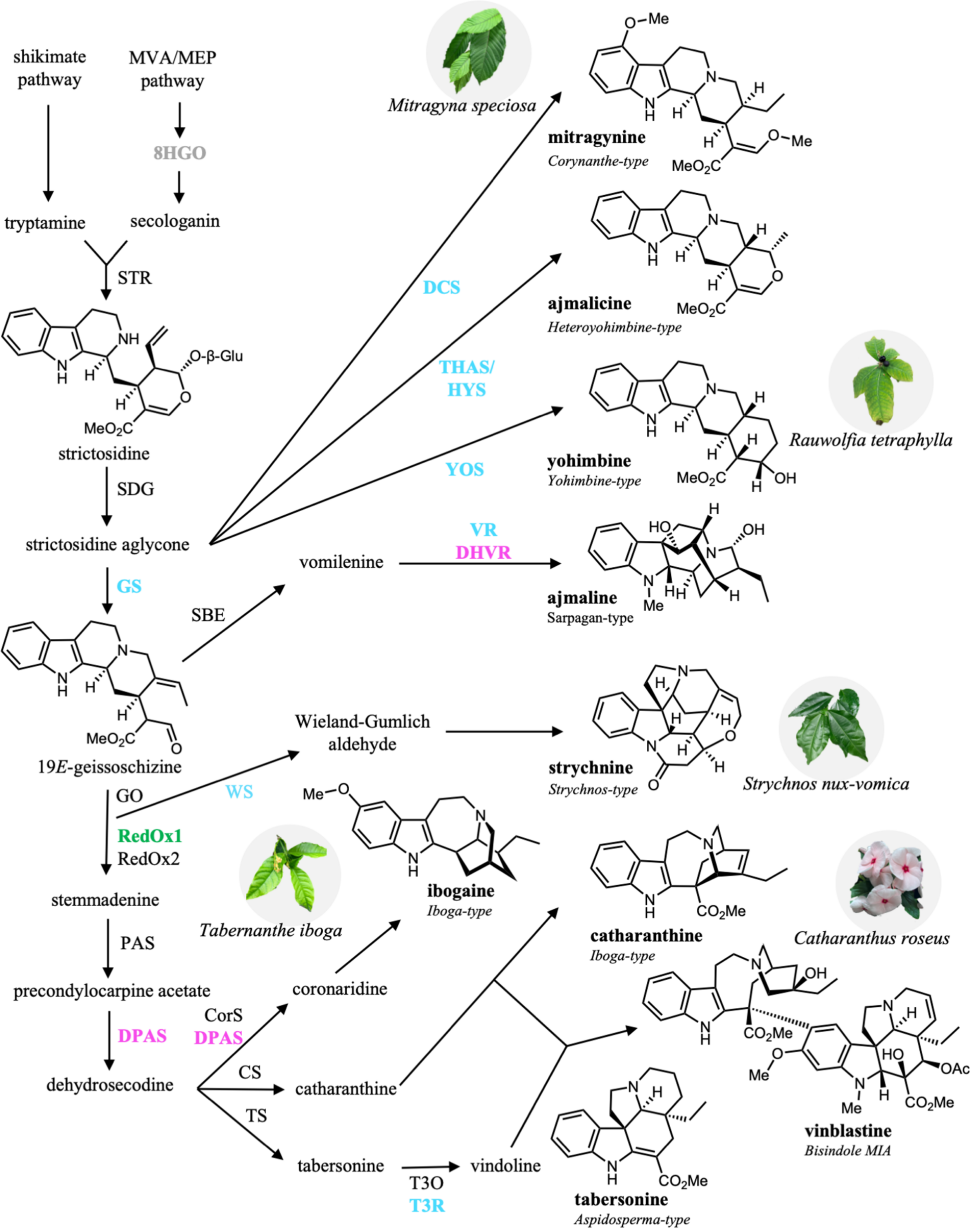
Roadmap to MDR-catalyzed reactions in MIA biosynthesis. A simplified diagram of the biosynthetic routes to major MIAs in various plants. MDRs conforming to the three major and one minor catalytic architectures described in the text are shown in gray (canonical), cyan (THAS-like), magenta (DPAS-like), and green (RedOx1-like). Additional biosynthetic enzymes are shown in black for reference. Abbreviations: 8-hydroxygeraniol oxidoreductase (8HGO), strictosidine synthase (STR), strictosidine β-d-glucosidase (SGD), geissoschizine synthase (GS), sarpagen bridge enzyme (SBE), dihydrocorynantheine synthases (DCS), tetrahydroalstonine synthase (THAS), heteroyohimbine synthase (HYS), yohimbine synthase (YOS), vomilenine reductase (VR), dihydrovomilenine reductase (DHVR), geissoschizine oxidase (GO), Weiland-Gumlich synthase (WS), reductive/oxidative enzyme 1 (RedOx1), reductive/oxidative enzyme 2 (RedOx2), precondylocarpine acetate synthase (PAS), dihydroprecondylocarpine acetate synthase (DPAS), coronaridine synthase (CorS), catharanthine synthase (CS), tabersonine synthase (TS), tabersonine 3-oxygenase (T3O), tabersonine 3-reductase (T3R).

The medicinal value of MIAs has fueled extensive efforts to elucidate the chemical and genetic basis of the biosynthesis of these molecules. As the biosynthetic pathways leading to many of MIA scaffolds have been elucidated, a notable pattern has emerged: only a limited number of enzyme families are involved in the biosynthesis of these molecules. One such family, the topic of this review, is the medium-chain dehydrogenases/reductase (MDR) family. MDRs comprise a large superfamily of oxidoreductase enzymes primarily involved in the reversible oxidation of alcohols and reduction of aldehydes. MDRs are found in all phyla of life and are further subclassified into 8 major groups including the cinnamyl alcohol dehydrogenase (CAD) family, enzymes that are typically involved in the oxidation and reduction of cinnamyl alcohols or aldehydes during lignin biosynthesis.^19–21^

MDRs share a highly conserved, ancient fold consisting of a *C*-terminal cofactor-binding (Rossman) domain and an *N*-terminal catalytic domain (Figure 2a,b). These enzymes are typically either dimeric or tetrameric.^22^ The active site is located in a large cleft at the interface of the *N*- and *C*-terminal domains. Zinc-dependent MDRs, which include members of the CAD family, typically coordinate two zinc ions: (i) a structural tetradentate zinc located in a loop in the catalytic domain, not in proximity to the active site, and (ii) a catalytic tridentate zinc located in the active site.^23^ The tridentate catalytic zinc coordination is completed by a water molecule that is displaced upon substrate binding^24^ (Figure 2c). Alternatively, tetradentate catalytic zinc coordination completed by a highly conserved glutamic acid has also been described.^25–27^ This alternative zinc coordination has been proposed to represent an intermediate conformation facilitating exchange of the zinc-coordinated water with the substrate oxygen. Reduction or oxidation is facilitated by the catalytic zinc through coordination to the oxygen of the alcohol or aldehyde substrate, enabling zinc to act as a Lewis acid during catalysis. A proton relay formed between an active site serine or threonine and the 2′O ribose of NADP­(H), and a histidine in the active site also contributes to promote acid/base catalysis^26^ (Figure 2b).

**Figure 2:**
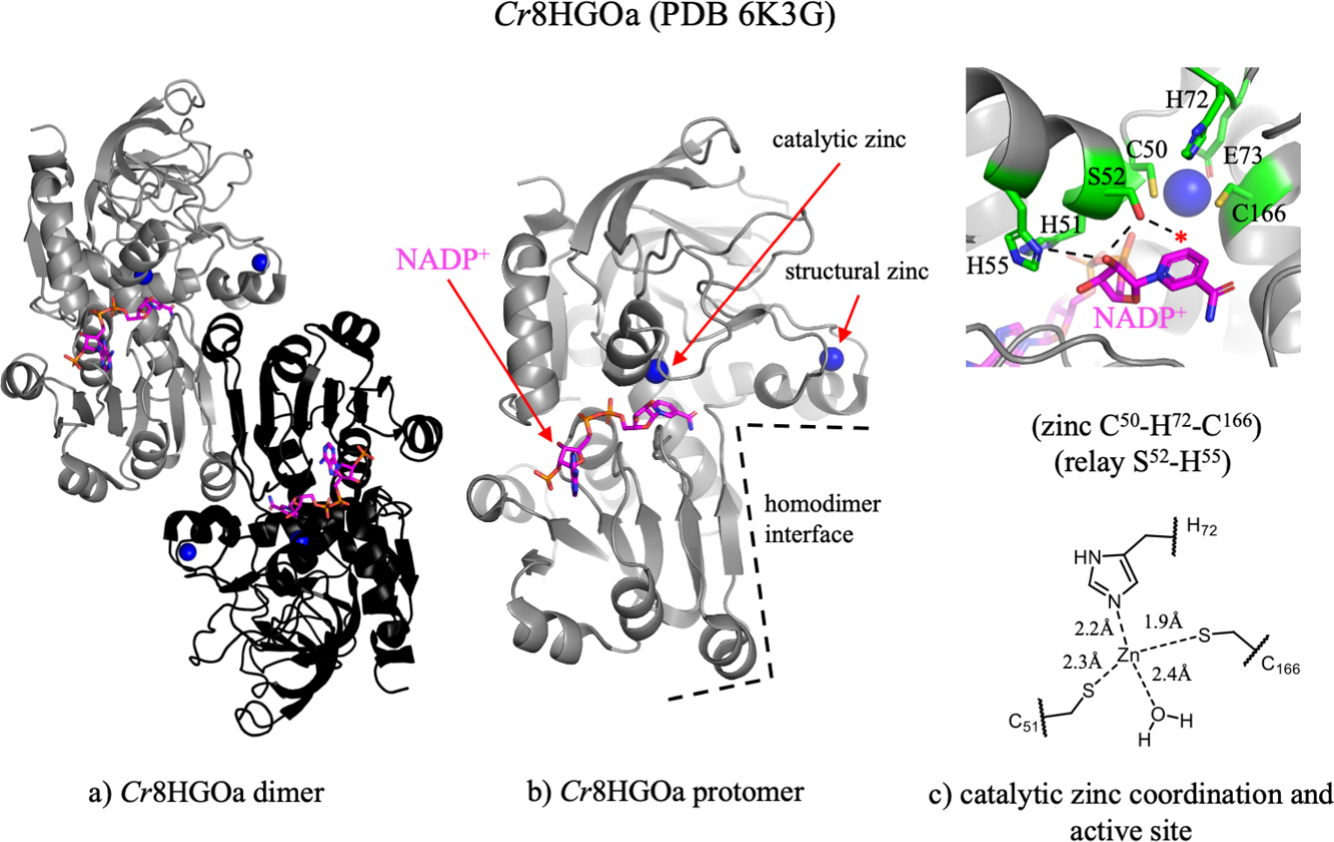
The MDR superfamily: Overall structure, active site, and catalytic zinc binding. (a) The overall fold of *Cr*8HGOa (PDB 6K3G)^18^ with dimer protomers shown in gray and black. (b) Single protomer of *Cr*8HGOa. The homodimer interface is marked with a dotted line. (c) The active site of *Cr*8HGOa and schematic of tridentate catalytic zinc coordination. Protein backbones are shown in gray, key residue sidechains in green (carbon), red (oxygen), blue (nitrogen) and yellow (sulfur), cofactor is shown in magenta, and zinc ions as blue spheres. Active site hydrogen bonding network is drawn with dotted lines and the zinc coordinating water molecule is represented as a red star. Bond distances of zinc coordination were measured in Pymol.

To date, at least 18 enzymatic steps in MIA biosynthesis have been shown to be catalyzed by MDRs. Notably, many of these steps involve the reductive quenching of iminium intermediates (Figure 1 and Table 1). All *except Rt*THAS5 and *Cr*8HGO are closely related to members of the CAD subfamily. Notably, many of these MDRs have undergone neofunctionalization of the active site to enable 1,2-reduction or 1,4-reduction of an iminium species, or 1,4-reduction of an α,β-unsaturated aldehyde species instead of 1,2-reduction of an aldehyde. The noncanonical catalytic activities of these MDRs are reflected by unique catalytic architectures, in some cases revealed through protein crystallography.^28^ Analogous to iminium reductase neofunctionalization from MDRs, in benzylisoquinoline alkaloid biosynthesis iminium reductase activity was shown to be neofunctionalized from aldo-keto reductases.^29,30^

**Table 1:** Characterized MDRs in MIA Biosynthesis

name	NCBI accession	reaction type	proposed substrate in MIA biosynthesis	references
Alstonia scholaris				
*As*GS	OM323326	1,2-iminium reductase	strictosidine aglycone	^31^
Camptotheca acuminata				
*Ca*8HGO	AY342355	alcohol oxidase	8-hydroxygeraniol	^32^
Catharanthus roseus				
*Cr*8HGO	KF302069	alcohol oxidase	8-hydroxygeraniol	^33^
*Cr*8HGOa	KF561458	alcohol oxidase	8-hydroxygeraniol	^34^
*Cr*THAS1	KM524258	1,2-iminium reductase	strictosidine aglycone	^35,36^
*Cr*THAS2	KU865323	1,2-iminium reductase	strictosidine aglycone	^35^
*Cr*THAS3	KU865322	1,2-iminium reductase	strictosidine aglycone	^35^
*Cr*THAS4	KU865324	1,2-iminium reductase	strictosidine aglycone	^35^
*Cr*HYS	KU865325	1,2-iminium reductase	strictosidine aglycone	^35^
*Cr*GS1	KF302079	1,2-iminium reductase	strictosidine aglycone	^37^
*Cr*GS2	KF302078	1,2-iminium reductase	strictosidine aglycone	^37^
*Cr*RedOx1	MF770509	1,2-iminium reductase	oxidized geissoschizine	^38^
*Cr*DPAS	KU865331	1,4-iminium reductase/1,4-α,β unsaturated aldehyde reductase	precondylocarpine acetate	^39^
*Cr*T3R	KP122966	1,2-iminium reductase	unnamed intermediate	^40^
*Cr*CAD^35^	KU865327	alcohol oxidase	cinnamaldehyde	
Cephalanthus occidentalis				
*Co*DCS	OQ129431	1,2-, 1,4-iminium reductase	strictosidine aglycone	^41^
Cinchona pubescens				
*Cp*DCS	MW456554	1,2-, 1,4-iminium reductase	strictosidine aglycone	^42^
*Cp*THAS1	PQ568391	1,2-iminium reductase	strictosidine aglycone	^43^
*Cp*THAS2	PQ568388	1,2-iminium reductase	strictosidine aglycone	^43^
Gardenia jasminoides				
*Gj*8HGO	OR065095	alcohol oxidase	8-hydroxygeraniol	^44^
Mitragyna parvifolia				
*Mp*DCS	OQ129432	1,2-, 1,4-iminium reductase	strictosidine aglycone	^41^
Mitragyna speciosa				
*Ms*DCS1	OQ129427	1,2-, 1,4-iminium reductase	strictosidine aglycone	^41,45,46^
*Ms*DCS2	OQ129428	1,2-, 1,4-iminium reductase	strictosidine aglycone	^41,45^
*Ms*DCS3	OQ129429	1,2-, 1,4-iminium reductase	strictosidine aglycone	^41^
*Ms*DCS4	OQ129430	1,2-, 1,4-iminium reductase	strictosidine aglycone	^41^
*Ms*THAS	OP800440	1,2-iminium reductase	strictosidine aglycone	^46^
*Ms*MDR4	OP800441	unknown	strictosidine aglycone	^46^
*Ms*HYS	OP800439	1,2-iminium reductase	strictosidine aglycone	^46^
Rauvolfia tetraphylla				
*Rt*THAS3	OR514625	1,2-iminium reductase	strictosidine aglycone	^47^
*Rt*THAS4A	OR514623	1,2-iminium reductase	strictosidine aglycone	^47^
*Rt*THAS4B	OR514624	1,2-iminium reductase	strictosidine aglycone	^47^
*Rt*THAS5	OR514622	1,2-iminium reductase	strictosidine aglycone	^47^
*Rt*YOS	OR514626	1,2-iminium reductase	strictosidine aglycone	^47^
*Rt*AMS	OR514628	1,2-iminium reductase	strictosidine aglycone	^47^
*Rt*GS	OR514629	1,2-iminium reductase	strictosidine aglycone	^47^
*Rt*MSTRG.5534	OR514630	unknown	19*E*-geissoschizine	^47^
*Rt*MSTRG.5531	OR514631	unknown	19*E*-geissoschizine	^47^
*Rt*MSTRG.5530	OR514632	unknown	19*E*-geissoschizine	^47^
*Rt*VR2 (DHVR)	KT369741	1,4-α,β unsaturated aldehyde reductase	19,20-dihydrovomilenine	^48^
Rauwolfia serpentina				
*Rs*GS-like	OQ591883			^49^
*Rs*VR2 (DHVR)	KT369740	1,4-α,β unsaturated aldehyde reductase	19,20-dihydrovomilenine	^48,49^
*Rs*DHVR	OQ591882	1,4-α,β unsaturated aldehyde reductase	19,20-dihydrovomilenine	^49^
*Rs*VR	OQ591881	1,2-iminium reductase	vomilenine	^49^
*Rs*CAD	KT369739	alcohol oxidase	cinnamaldehyde	^48^
Strychnos nux-vomica				
*Snv*WS	OM304294	1,2-iminium reductase	18-OH norfluorocurarine	^50^
Strychnos potatorum				
*Sp*WS	OM304302	1,2-iminium reductase	18-OH norfluorocurarine	^50^
Tabernanthe iboga				
*Ti*DPAS1	MK840855	1,4-iminium reductase/1,4-α,β unsaturated aldehyde reductase	precondylocarpine acetate	^51^
*Ti*DPAS2	MK840856	1,4-iminium reductase/1,4-α,β unsaturated aldehyde reductase	precondylocarpine acetate	^51^

This review first summarizes the currently characterized MIA biosynthetic MDRs. We then present a mechanistic framework based on these documented activities to enable continued discovery and investigation of this important family of enzymes.

## Medium-Chain Dehydrogenase/Reductases (MDRs) in MIA Biosynthesis

### Canonical Redox Reaction: 8-Hydroxygeraniol Oxidoreductase (8HGO)

MIAs are derived from the iridoid monoterpene secologanin.^52^ An early step in secologanin biosynthesis is the double oxidation of the dialcohol 8-hydroxygeraniol to the dialdehyde 8-oxogeranial, a reaction catalyzed by a canonical MDR named 8HGO (also called 10HGO due to a discrepancy in carbon numbering convention)^53^ (Figure 3a). The enzyme can oxidize the C1 and the C8 alcohol moieties in either order. Orthologues of 8HGO have been characterized in (*Ca*8HGO),^32^ (*Gj*8HGO),^44^ and (*Cr*8HGO^33^ and *Cr*8HGOa^34^ with 52% sequence identity). *Cr*8HGOa was suggested to be the primary enzyme in the oxidation of 8-hydroxygeraniol in based on its preference for 8-hydroxygeraniol compared to other terpene substrates.^34^ However, single cell transcriptomics shows that only *Cr*8HGO is highly coexpressed with the other iridoid biosynthetic enzymes in IPAP cells.^54,55^

**Figure 3:**
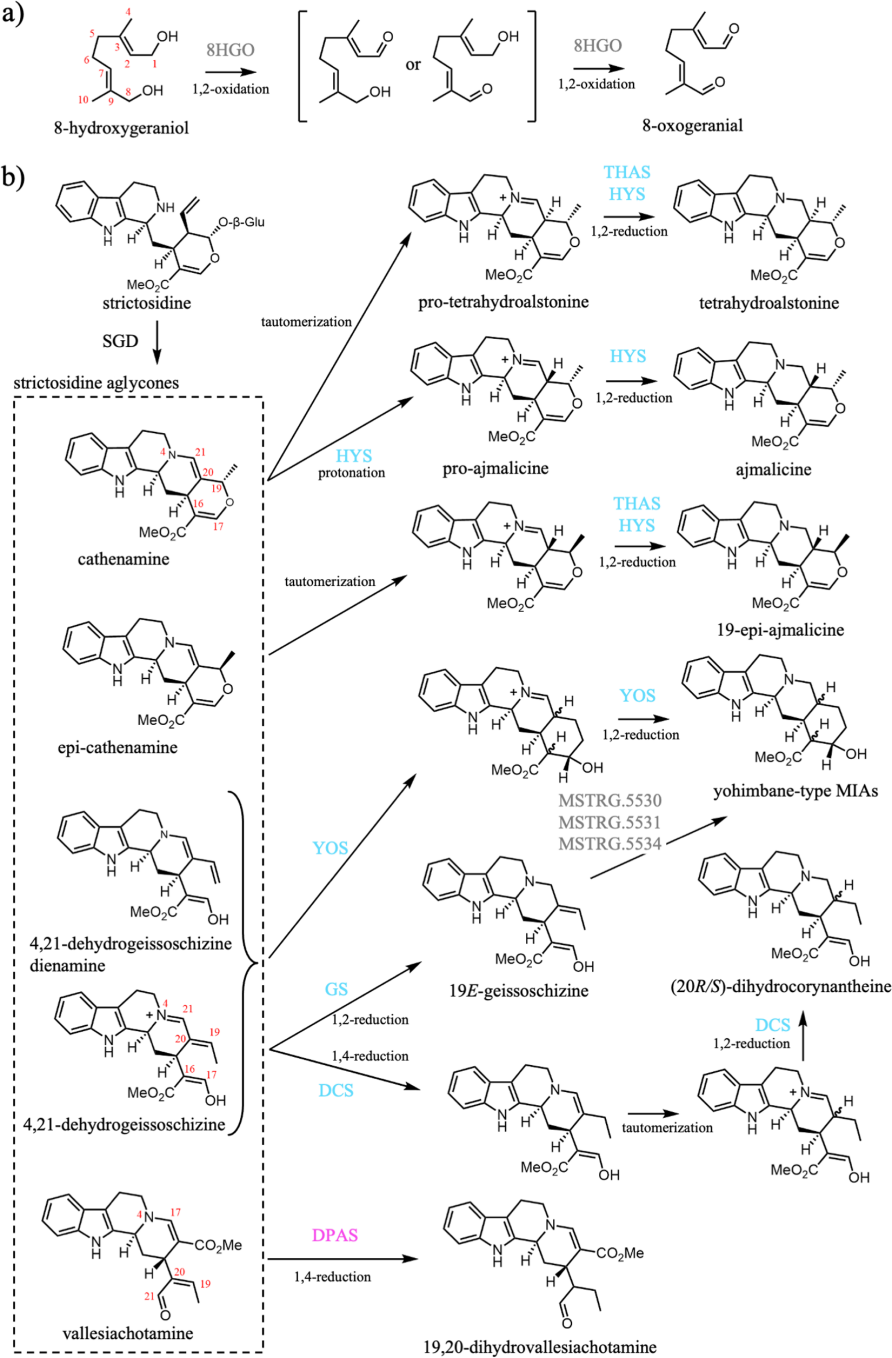
Early MIA pathway MDR-catalyzed reactions. (a) 8HGO catalyzed oxidation of 8-hydroxygeraniol. (b) MDR-catalyzed reductive trappings of strictosidine aglycone by DPAS, THAS/HYS, DCS, GS, and YOS. MDRs are highlighted based on their catalytic architectures described in the text: gray (canonical), cyan (THAS-like), magenta (DPAS-like), and green (RedOx1-like). Abbreviations: 8-hydroxygeraniol oxidoreductase (8HGO), strictosidine β-d-glucosidase (SGD), geissoschizine synthase (GS), dihydrocorynantheine synthases (DCS), tetrahydroalstonine synthase (THAS), heteroyohimbine synthase (HYS), yohimbine synthase (YOS), dihydroprecondylocarpine acetate synthase (DPAS).

### Reduction of the Iminium Moiety of Strictosidine Aglycone

Strictosidine, formed by the condensation of tryptamine and secologanin, marks the first alkaloid intermediate in MIA biosynthesis (Figure 1). Deglycosylation of strictosidine yields a reactive species that can undergo cyclizations that result in formation of iminium ions. These iminium intermediates are subsequently reduced by a range of MDRs to form several distinct alkaloids (Figure 3).^31,35–37,41,42,45,47,56,57^ It is not clear whether the isomerization and cyclization of strictosidine aglycone is controlled by these reductases or some other factor.

#### Heteroyohimbine Synthases (THAS and HYS) (1,2-Iminium Reduction)

Two consecutive cyclizations of strictosidine aglycone results in formation of the heteroyohimbine-scaffold cathenamine (C19*S*) and epi-cathenamine (C19*R*). The enamine of cathenamine and epi-cathenamine likely tautomerizes to the corresponding iminium species, which can be reductively trapped by *Cr*THAS1,2,3 and *Cr*HYS in^35,36,58–60^ (Figure 3). *Cr*THAS1-4, demonstrate variable stereoselectivity in the reduction of cathenamine (19*S*) and epi-cathenamine (19*R*) to form tetrahydroalstonine (19*S*, 20*S*) and 19-*epi*-ajmalicine (19*R*, 20*R*), respectively. In these cases, protonation at C20 occurs with the same stereochemistry as the previously set C19. *Cr*HYS demonstrates additional 19*S*, 20*R* stereoselectivity and generates three stereoisomers from cathenamine and *epi*-cathenamine to form a mixture of tetrahydroalstonine (19*S*, 20*S*), 19-*epi*-ajmalicine (19*R*, 20*R*) and ajmalicine (19*S*, 20*R*). THAS/HYS have been reported not only in (*Cr*THAS1,2,3 and *Cr*HYS), but also in (*Rt*THAS3, *Rt*THAS4A, *Rt*THAS4B, *Rt*THAS5, *Rt*AMS (HYS-like)),^47^ (*Ms*THAS, and *Ms*HYS),^46^ and (*Cp*THAS1 and 2).^43^ *Cr*THAS1 and 2 have additionally been shown to catalyze reduction of a late stage iminium intermediate leading to anhydrovinblastine.^54^ Reduction of strictosidine aglycone to tetrahydroalstonine also occurs as a minor product in many of the enzymatic reactions described below (GS, DCS, YOS, WS).^41,47,61^ This could be the result of a natural prevalence for tetrahydroalstonine from strictosidine aglycone, a hypothesis supported by the fact that tetrahydroalstonine is also the primary product formed from chemical reduction of strictosidine aglycone by sodium borohydride.^58^ This observation suggests that tetrahydroalstonine may have been the first strictosidine-derived alkaloid to have evolved, but additional evidence is needed to support this hypothesis.

#### Dihydrocorynantheine Synthases (DCS) (1,2- and 1,4-α,β-Unsaturated Iminium Reduction)

A single cyclization of strictosidine aglycone leads to the intermediate 4,21-dehydrogeissoschizine, which possesses a conjugated 1,4-iminium and can therefore undergo both 1,2- and 1,4-iminium reductions. Dihydrocorynantheine, a central precursor to kratom and cinchona-derived alkaloids, is produced by the consecutive 1,4- and 1,2-iminium reductions of 4,21-dehydrogeissoschizine (Figure 3). Dihydrocorynantheine synthase (DCS) catalyzes these reductions and was first identified in , *Cp*DCS. *Cp*DCS catalyzes the double reduction of 4,21-dehydrogeissoschizine to (20*R*)-dihydrocorynantheine, an intermediate to dihydro-series quinine related alkaloids.^42^ *Cp*DCS also reduces 5-methoxylated strictosidine aglycone to form the corresponding methoxylated (20*R*)-dihydrocorynantheine species.^43^ The discovery of *Cp*DCS led to further discovery and characterization of orthologues in (*Ms*DCS1–4), (*Mp*DCS), and (*Co*DCS).^41,45^ While the DCS orthologues *Cp*DCS, *Ms*DCS1–4, *Mp*DCS, and *Co*DCS reduce strictosidine aglycone to (20*R*)-dihydrocorynantheine, *Ms*DCS1, *Mp*DCS, and *Co*DCS additionally produce (20*S*)-dihydrocorynantheine.^41,45^ Notably, kratom produces alkaloids derived from both 20*S* (mitragynine) and 20*R* (speciogynine) dihydrocorynantheine stereoisomers, explaining why DCS homologues with differing stereoselectivity are found in this plant. The control of stereoselectivity at C20 is proposed be due to the mode of binding of 4,21-dehydrogeissoschizine that is impacted by changes in the enzyme binding pocket shape.^45^

#### Geissoschizine Synthase (GS) (1,2-Iminium Reduction)

Geissoschizine synthase (GS) catalyzes 1,2-reduction of the iminium moiety of 4,21-dehydrogeissoschizine to form 19*E*-geissoschizine^37,56^ (Figure 3). Although GS and DCS share the same substrate, DCS catalyzes sequential 1,4- and 1,2-iminium reductions^41^ while GS catalyzes a single 1,2-iminium reduction.^37,56^ Two homologues, GS1 and GS2, have been reported in , sharing 91% sequence identity,^37^ and additional orthologues have been reported in^47^ and .^31^ *Cr*GS has also been shown to catalyze the downstream 1,4-iminium reduction of precondylocarpine acetate, though at low catalytic efficiency.^56^

#### Yohimbine Synthases (YOS) (1,2-Iminium Reduction)

Biosynthesis of yohimbine-type MIAs in may occur by two distinct biosynthetic routes^47^ (Figure 3). In one route, a single enzyme, yohimbane synthase (*Rt*YOS), catalyzes the cyclization of the enamine or iminium form of 4,21-dehydrogeissoschizine to produce an iminium intermediate that is then reduced to form the yohimbane stereoisomers rauwolscine, yohimbine, and coryanthine. The second route likely proceeds via reduction of the iminium of 4,21-dehydrogeissoschizine by GS to form (19*E*)-geissoschizine. Subsequent reduction and cyclization are then catalyzed by a second MDR, MSTRG.5530, 5531, or 5534. *Rt*GS, MSTRG.5530, 5531, and 5534 are clustered in the genome.

#### Parallels in Ipecac Alkaloid Biosynthesis

Notably, analogous reductions of aglycone intermediates occur during the biosynthesis of ipecac alkaloids in and . These MIA related alkaloids are biosynthesized by the condensation of secologanin (or secologanic acid) and dopamine, in place of tryptamine. Deglycosylation of the condensation product forms an unstable aglycone which cyclizes to form iminium intermediates that are reductively trapped by MDRs.^62^ These MDRs were proposed to have evolved in parallel to the corresponding MIA enzymes.

### Downstream Reductases

#### RedOx1 (1,2-Iminium Reduction)

In the biosynthesis of iboga- and aspidosperma-type alkaloids, geissoschizine is converted to the intermediate stemmadenine through the concerted action of the cytochrome P450 geissoschizine oxidase (GO), MDR RedOx1, and the alpha-keto reductase RedOx2 (Figure 4a).^38^ The unstable nature of these intermediates precludes direct identification of the substrate and product of RedOx1. However, through the investigation of spontaneous rearrangements to stable intermediates and inspection of metabolite profiles that are formed upon silencing of these genes, RedOx1 from has been proposed to catalyze a 1,2-iminium reduction of the immediate product of GO, dihydropreakuammicine.

**Figure 4:**
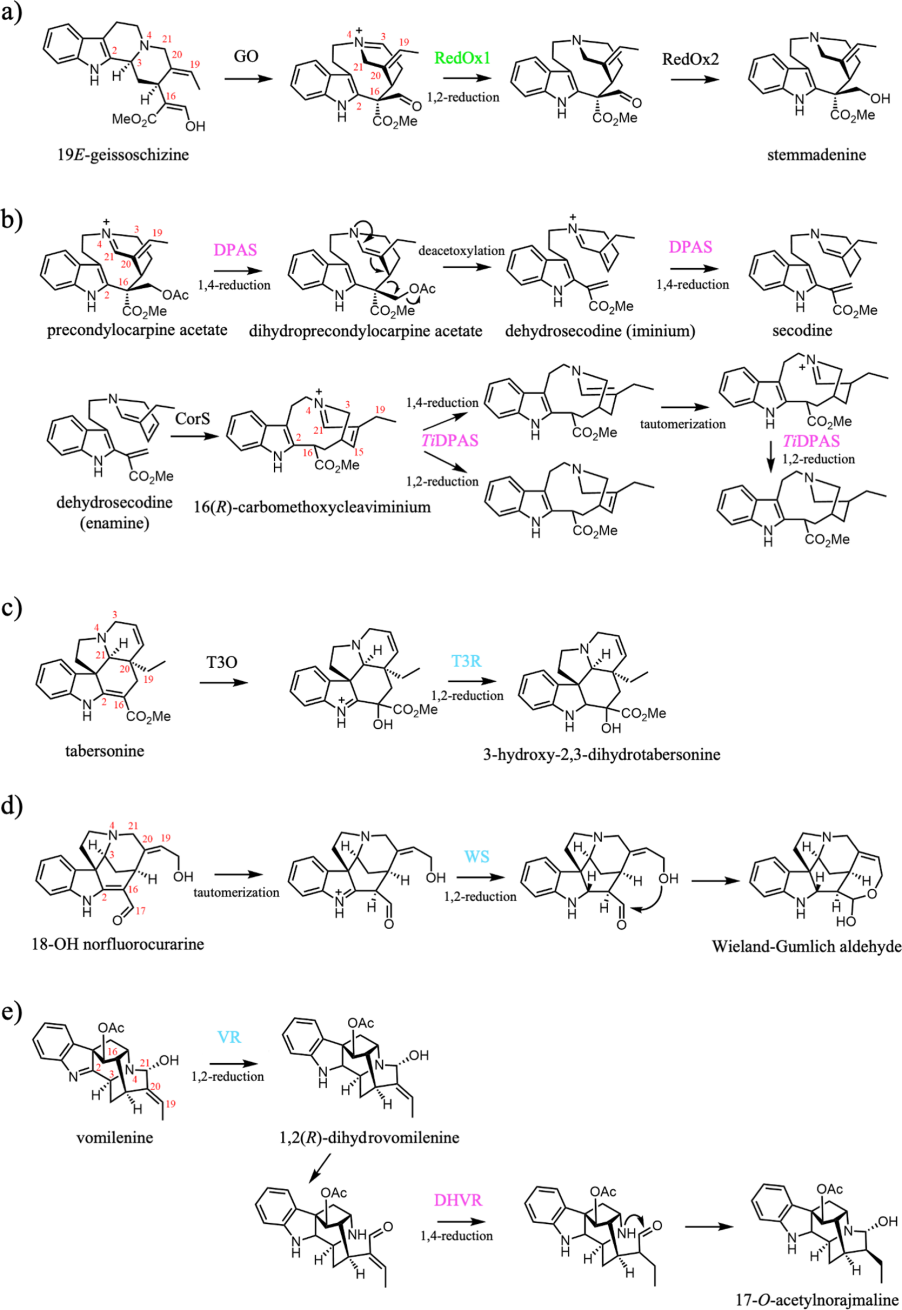
Downstream MDR-catalyzed reductions. (a) Concerted action of GO, RedOx1, and RedOx2 in the conversion of 19*E*-geissoschizine to stemmadenine. (b) DPAS catalyzed reductions of precondylocarpine acetate and downstream intermediates. (c) Concerted action of T3O and T3R in the conversion of tabersonine to 3-hydroxy-2,3-dihydrotabersonine. (d) WS catalyzed reductions of 18-OH norfluorocarine. (e) VR and DHVR catalyzed reductions of vomilenine and 1,2­(*R*)-dihydrovomilenine. MDRs are highlighted based on their catalytic architectures described in the text: gray (canonical), cyan (THAS-like), magenta (DPAS-like), and green (RedOx1-like). Abbreviations: vomilenine reductase (VR), dihydrovomilenine reductase (DHVR), geissoschizine oxidase (GO), Weiland-Gumlich synthase (WS), reductive/oxidative enzyme 1 (RedOx1), reductive/oxidative enzyme 2 (RedOx2), dihydroprecondylocarpine acetate synthase (DPAS), coronaridine synthase (CorS), tabersonine 3-oxygenase (T3O), tabersonine 3-reductase (T3R).

#### Dihydroprecondylocarpine Synthase (DPAS) (1,4-α,β-Unsaturated Iminium and 1,4-α,β-Unsaturated Aldehyde Reduction)

Further downstream in the biosynthesis of iboga- and aspidosperma-type alkaloids, the dihydropreakuammicine-derived intermediate stemmadenine is acetylated and then oxidized to form precondylocarpine acetate. Dihydroprecondylocarpine acetate synthase (DPAS) catalyzes the 1,4-iminium reduction of precondylocarpine acetate to form dihydroprecondylocarpine acetate, which then spontaneously deacetoxylates to form dehydrosecodine^28,39^ (Figure 4b). Although precondylocarpine acetate and dehydrosecodine are unstable, the structure of these two putative intermediates has been inferred from the stable degradation product, angryline.^28^ DPAS has been characterized in^39^ and two homologues have been characterized in , *Ti*DPAS1 and *Ti*DPAS2, which share ∼85% sequence identity to *Cr*DPAS.^51^ Assays using isotopically labeled NADPH confirm the catalysis of 1,4-iminium reduction by and DPAS by the observed deuterium labeling at C19 of dihydroprecondylocarpine acetate, subsequently detected in angryline.^39,63^ Dehydrosecodine is then cyclized by tabersonine synthase (TS), catharanthine synthase (CS), or coronaridine synthase (CorS) to generate three distinct structural scaffolds.^28,39,51,63^ *Cr*DPAS was shown to additionally reduce dehydrosecodine (iminium form) to secodine by a second, nonstereoselective, 1,4-iminium reduction. Secodine then cyclizes to vincadifformine^28,64^ (Figure 4b). *Ti*DPAS1 and 2 additionally catalyze iminium reduction of the downstream biosynthetic intermediate, 16­(*R*)-carbomethoxycleaviminium, that is the immediate precursor for (−)-coronaridine, pseudotabersonine, and pseudovincadifformine.^63^ *Cr*DPAS can also reduce strictosidine aglycone through a 1,4-α,β-unsaturated aldehyde reduction at C19 to from 19,20-dihydrovallesiachotamine^28^ (Figure 3b), revealing that DPAS can catalyze both 1,4-iminium and 1,4-α,β-unsaturated aldehyde reductions.

#### Tabersonine 3-Reductase (T3R) (1,2-Iminium Reduction)

Tabersonine 3-reductase (T3R) from catalyzes a reduction step in the biosynthesis of the aspidosperma-type MIA vindoline.^40^ Tabersonine is converted to 3-hydroxy-2,3-dihydrotabersonine by concerted action of tabersonine-3-oxidase (T3O) and T3R (Figure 4c). T3O most likely generates an epoxide functionality from the alkene of tabersonine. The electron rich amine moiety adjacent to this epoxide would cause the spontaneous opening of this epoxide through formation of an iminium moiety, which could then be reduced by T3R.^40,65^

#### Weiland-Gumlich Synthase (WS) (1,2-Iminium Reduction)

Weiland-Gumlich synthase (WS) was first identified in and is involved in strychnine biosynthesis.^50^ *Snv*WS catalyzes the reduction of the imine form of the enamine in 18-OH norfluorocurarine to yield the Wieland-Gumlich aldehyde (Figure 4d), a key proposed intermediate in strychnine biosynthesis.^66^ WS can additionally, albeit less efficiently, catalyze reduction of norfluorocurarine prior to C18-hydroxylation. Similar to proposed THAS/HYS catalyzed enamine reduction, the *Snw*WS 2,16-double bond reduction is proposed to be facilitated by protonation of the enamine moiety to form an indole iminium which can be readily reduced by NADPH.^50^ An orthologue of *Snw*WS was identified in (*Sp*WS) sharing 93% amino acid sequence identity showing a similar catalytic profile.^50^

#### Vomilenine Reductases (VR and DHVR) (1,2-Imine Reduction, VR; 1,4-α,β-Unsaturated Aldehyde Reduction, DHVR)

Biosynthesis of sarpagan-type MIAs involve the oxidation of geissoschizine by sarpagan bridge enzyme (SBE),^67^ instead of oxidation by GO as is the case for strychnos-, iboga-, and aspidosperma-type MIAs. The initial oxidation product produced by SBE, polyneuridine aldehyde, is derivatized to form vomilenine. Two MDRs, vomilenine reductase (VR) and dihydrovomilenine reductase (DHVR), then catalyze consecutive reduction reactions on this intermediate. These enzymes were first detected in cell cultures^68,69^ and later cloned and characterized.^48,49^ First, VR catalyzes 1,2-imine reduction of vomilenine to form 1,2­(*R*)-dihydrovomilenine. Second, DHVR catalyzes a 1,4-reduction of the conjugated double bond (position 19,20) of the VR product to form 17-*O*-acetylnorajmaline^48,49,68,69^ (Figure 4e). A truncated form of DHVR (named VR2) may also catalyze 19,20-reduction of vomilenine in and .^68,69^ The reduction of the 19,20-double bond likely proceeds via a 1,4-α,β-unsaturated aldehyde intermediate, which is generated by ring opening at N4–C21 to form an aldehyde. This conjugated aldehyde undergoes a 1,4-α,β-unsaturated aldehyde reduction, which is catalyzed by DHVR, and reformation of the C4–C21 bond reconstitutes the original ring (Figure 4e).

## Mechanisms of MDRs

With the discovery of MDRs that catalyze a wide range of enzymatic reductions, it is now possible to explore the mechanistic basis by which the canonical MDR active site can be modulated to expand the repertoire of possible reduction reactions.

### Canonical Reduction

Of all the MDRs involved in MIA biosynthesis, only 8HGO catalyzes the typical oxidation or reduction of an alcohol or aldehyde (Figure 5). 8HGO is mechanistically and structurally well-characterized (*Cr*8HGOa (PDB 6K3G, 6KJ5)).^18^ *Cr*8HGOa displays an active site architecture that is typical of MDRs in which the catalytic zinc is coordinated in the canonical manner by residues C50, H72, C166, along with a water molecule.^18^ Binding of 8-hydroxygeraniol displaces this water molecule, enabling the coordination of the hydroxyl group at C1 or C8 to zinc. S52 deprotonates the hydroxyl group through a proton relay with the 2′O ribose of NADP^+^ and H55, thereby allowing hydride abstraction by NADP^+^ from carbon to generate the aldehyde. In *Cr*8HGO, H55 is substituted to a leucine, suggesting a functional proton relay may form with a different residue or a water molecule.^18,28^ Notably, oxidation of 8-hydroxygeraniol to 8-oxogeranial requires two sequential oxidations that appear to proceed in no specific order. This requires multiple binding conformations of the substrate to correctly position both ends in the correct orientation for efficient alcohol deprotonation and hydride release to NADP^+^. This is likely facilitated by the conformational flexibility of this linear substrate.

**Figure 5:**
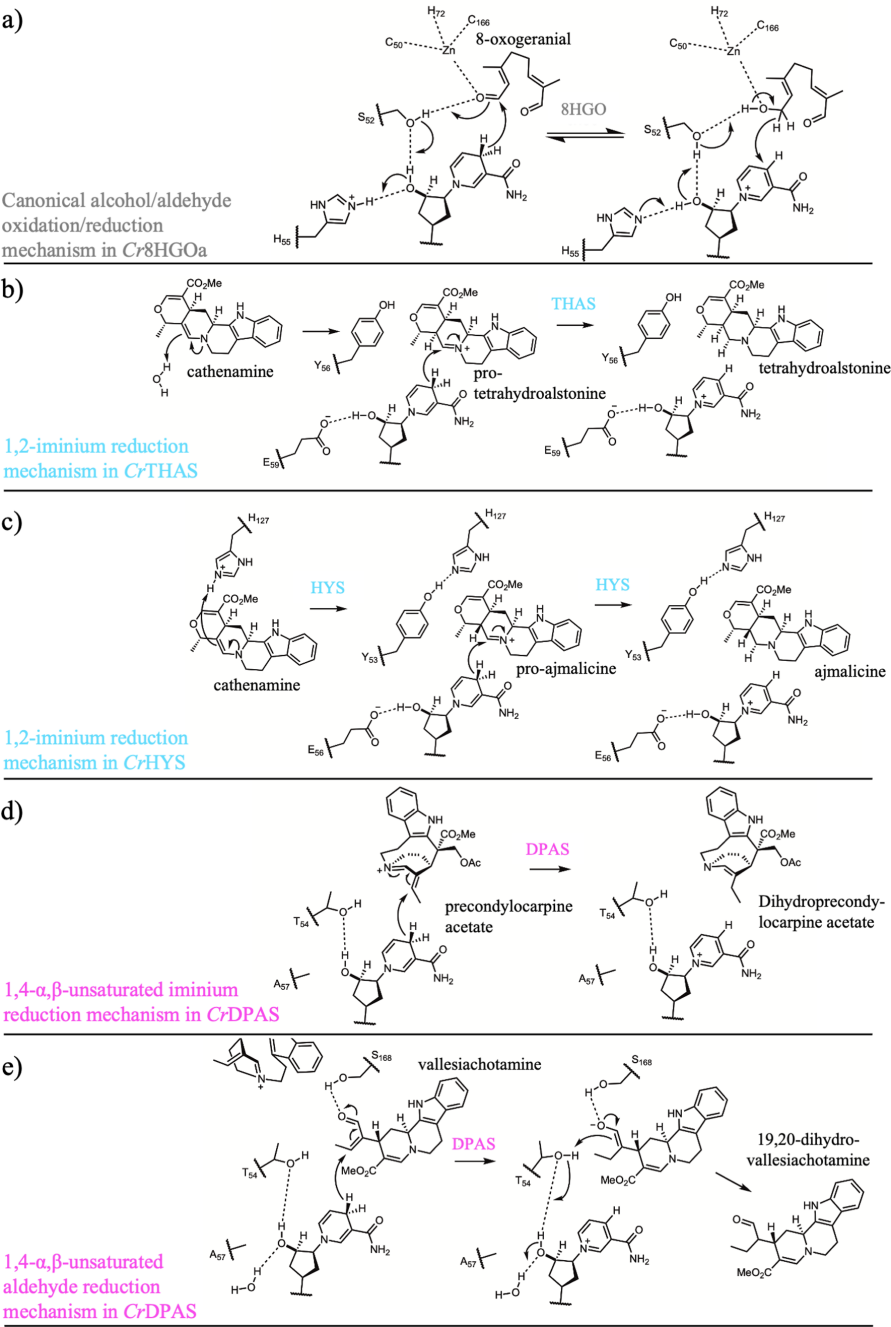
Proposed mechanisms of MDR-catalyzed reductions of 1,2-, 1,4-α,β-unsaturated iminium, 1,4-α,β-unsaturated aldehyde and 1,2-carbonyl moieties. (a) Canonical alcohol/aldehyde oxidation/reduction mechanism in *Cr*8HGOa. (b,c) 1,2-Iminium reduction mechanism in *Cr*THAS (b) and *Cr*HYS (c). (d) 1,4-α,β-Unsaturated iminium reduction mechanism in *Cr*DPAS. (e) 1,4-α,β-Unsaturated aldehyde reduction mechanism in *Cr*DPAS. Abbreviations: 8-hydroxygeraniol oxidoreductase (8HGO), tetrahydroalstonine synthase (THAS), heteroyohimbine synthase (HYS), dihydroprecondylocarpine acetate synthase (DPAS).

### 1,2-Iminium Reduction

The reduction of iminium moieties is observed throughout MIA biosynthesis. In all known cases, these iminium species are reduced by MDRs with a modified active site architecture. Iminium reductases THAS, THAS orthologues and GS, which have each been structurally characterized by protein crystallography, have an active site with distinct changes compared to 8HGO (*Cr*THAS1 (PDB 5FI3, 5FI5), *Cr*THAS2 (PDB 5H82, 5H81), *Cr*HYS (PDB 5H83),^35^ *Cr*GS (PDB 8A3N)^28^). Instead of tridentate coordination to zinc, these proteins have tetradentate coordination in which a nearby glutamic acid, not water, completes the coordination sphere (C51, H73, E74, and C168 (GS numbering)) (Figure 6). This alternative zinc coordination has been reported in MDRs and is suggested to represent an intermediate conformation that facilitates exchange of the tridentate water for the substrate oxygen.^25–27^ However, it seems unlikely that the positively charged iminium substrate could displace this glutamic acid to coordinate to the zinc cofactor. We propose that the tetradentate coordination observed in all reported crystal structures of *Cr*THAS1, *Cr*THAS2, *Cr*HYS, and *Cr*GS represents the primary mode of catalytic zinc coordination for these enzymes. Site-directed mutagenesis of *Cr*GS H73 and C168 resulted in complete loss of activity. Although this could suggest a catalytic role for zinc,^28^ a more likely explanation is that this zinc plays a critical role in maintaining the structure of the active site.

**Figure 6:**
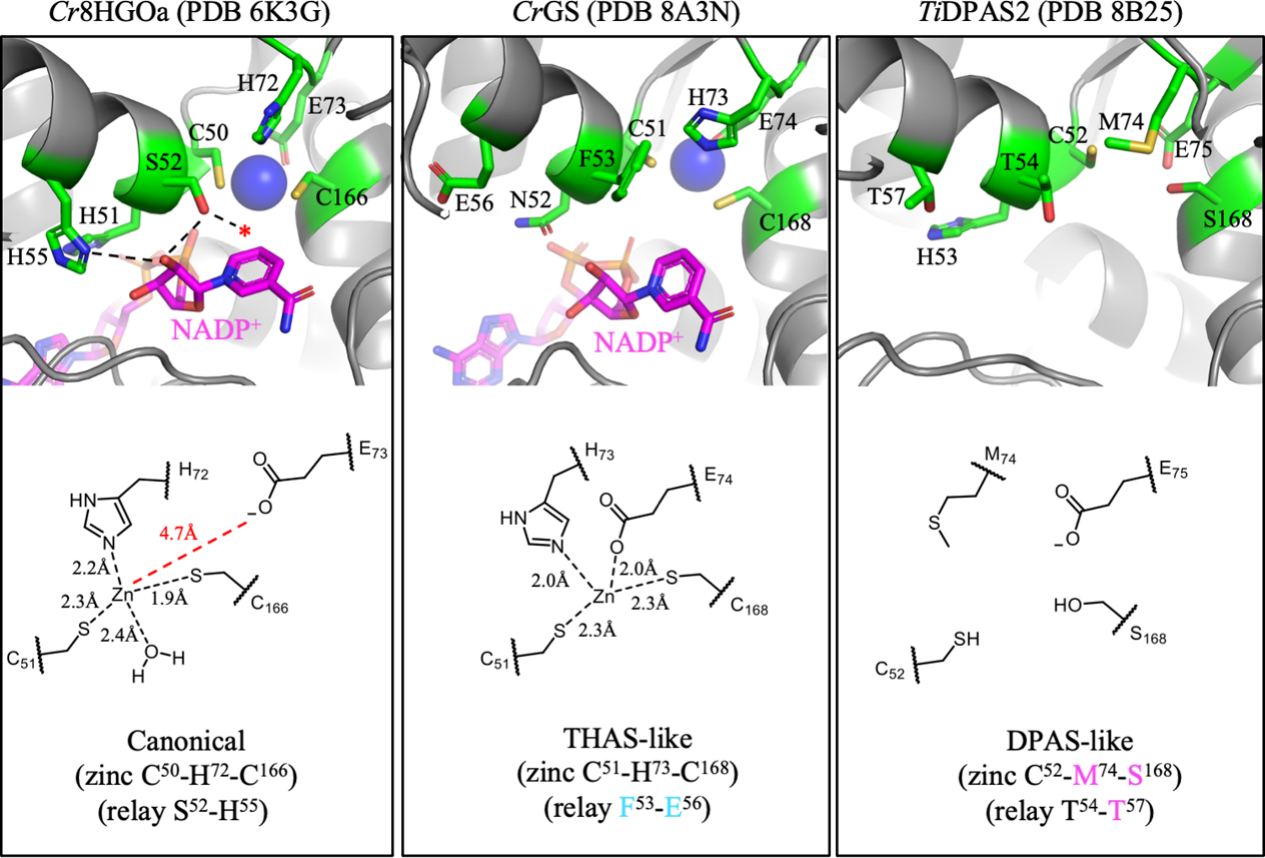
The three major catalytic architectures of MIA MDRs. The active sites of representative MIA MDRs for canonical (*Cr*8HGOa; PDB 6K3G),^18^ THAS-like (*Cr*GS; PDB 8A3N),^28^ and DPAS-like (*Ti*DPAS2; PDB 8B25).^28^ Protein backbones are shown in gray, key residue sidechains in green (carbon), red (oxygen), blue (nitrogen) and yellow (sulfur), cofactor is shown in magenta, and zinc ions as blue spheres. Hydrogen bonding networks are marked by dotted lines and the zinc coordinating water molecule is represented by a red star. Corresponding schematics of zinc coordination for each respective architecture are shown below. Distances were measured in Pymol.

THAS, THAS orthologues and GS also appear to have a disrupted proton relay, since the histidine that normally serves this role is not conserved. Additionally, the catalytic acid/base residue (position 52, normally a serine or threonine) is always substituted with an aromatic residue (tyrosine, phenylalanine, or tryptophan). The disruption of the proton relay, the lack of the active site serine or threonine, combined with the more reactive nature of iminium substrates, suggest that these enzymes likely catalyze iminium reduction by the proper coordination of substrate relative to the NADPH cofactor without participation of additional active site residues or zinc^28,35,38,45^ (Figure 5).

MDRs with these active site features—herein termed THAS-like—include THAS, HYS, GS, DCS, YOS, T3R, VR, and WS. Several THAS-like MDRs likely require substrate protonation prior to iminium reduction (DCS, THAS, HYS, WS, VR) to generate an iminium ion from the corresponding, less reactive enamine or imine species. Only in *Cr*HYS has an active site residue (H127) been proposed to carry out this protonation, whereas for *Cr*THAS1, 2, *Ms*DCS, and *Cp*CDS tautomerization may occur spontaneously in solution or be mediated by an active site water molecule in the absence of a properly positioned side chain.^35,45^ The protonation of enamine substrates prior to iminium reduction sets the stereochemistry at the respective chiral carbon based on the face at which protonation occurs. This is most likely facilitated by (i) the orientation of the substrate in the binding pocket and the location of the protonating side chain or bound water molecule, or (ii) steric properties of the substrate impacting the availability of the face for protonation. In *Cr*HYS, H127 was demonstrated to be responsible for 20*R* stereochemistry in ajmalicine, while in *Cr*THAS, a water molecule was proposed to generate 20*S* stereochemistry in tetrahydroalstonine.^35^ The combinatorial effect of several binding pocket residues was shown to control the C20 stereochemistry during the reduction of 4,21-dehydrogeissoschizine to (20*R*) or (20*S*)-dihydrocorynantheine by *Ms*DCS and *Cp*DCS through changes in substrate orientation in the binding pocket.^45^ Interestingly, the (20*S*)-dihydrocorynantheine producing DCS orthologues *Ms*DCS1, *Mp*DCS, and *Co*DCS, in contrast to all other THAS-like MDRs, each retain the catalytic acid/base serine or threonine. It is possible that this residue is responsible for the stereoselective protonation of 4,21-dehydrogeissoschizine, though this has not been shown.

RedOx1 also catalyzes a 1,2-iminium reduction, but this enzyme does not share the THAS-like active site features. Instead, the active site architecture of RedOx1 closely resembles that of 8HGO, with the exception of a single mutation in the catalytic zinc binding site, C166G (*Cr*8HGOa numbering). This suggests that the binding of catalytic zinc is also disrupted in RedOx1. The RedOx1-like active site architecture is, to date, unique among known MDRs. This enzyme may follow a mechanism similar to THAS-like MDRs.

### 1,4-α,β-Unsaturated Iminium Reduction

DPAS, which catalyzes 1,4-reduction of an iminium species (precondylocarpine acetate), likely follows a similar mechanism to the one proposed for THAS-like MDRs (Figure 5). However, there are several key unique features in the active site of DPAS. Most notably, *Cr*DPAS and *Ti*DPAS2 crystal structures show that the catalytic zinc is missing entirely, due to substitutions at the zinc coordination sphere (positions 72 and 166, *Cr*8HGOa numbering) (Figure 6). The loss of the catalytic zinc may expand the size of the binding pocket to accommodate larger substrates.^28^ Although the catalytic acid/base residue is conserved as a serine or threonine in DPAS homologues, mutagenesis of *Cr*DPAS demonstrated that this residue is not crucial for iminium reduction. Additionally, the H55 that is required for a functional proton relay is not conserved in DPAS homologues suggesting that this residue is not essential for catalytic activity in *Cr*DPAS. Moreover, mutation of this residue in *Cr*DPAS had no impact on catalysis. Reintroduction of the catalytic zinc coordinating residues by mutagenesis also had no effect on *Cr*DPAS activity.^28^

### 1,4-α,β-Unsaturated Aldehyde Reduction

DPAS can also catalyze 1,4-α,β-unsaturated aldehyde reduction as demonstrated by the reduction of strictosidine aglycone to 19,20-dihydrovallesiachotamine (Figure 3b). Mutagenesis suggests that the canonical catalytic acid/base residue T54, which is not required for 1,4-iminium reduction of the precondylocarpine iminium species, plays a crucial role in this 1,4-α,β-unsaturated aldehyde reduction.^28^ This led to a proposed mechanism in which hydride attack on the strictosidine aglycone isomer vallesiachotamine reduces the 19,20-double bond, forming an oxyanion intermediate. Residue S168 (166 in *Cr*8HGOa), conserved in *Cr*DPAS, *Ti*DPAS1/2, and *Rs*DHVR (Figure 7), is near the catalytic threonine/serine and may act to stabilize the oxyanion intermediate (Figure 5). The oxyanion is likely quenched through reformation of the original aldehyde while the adjacent double bond is protonated (Figure 5). T54 likely plays a crucial catalytic role through protonation,^28^ possibly forming a proton relay with the 2′O ribose of NADPH. This mechanism also likely applies to DHVR/VR2.

**Figure 7:**
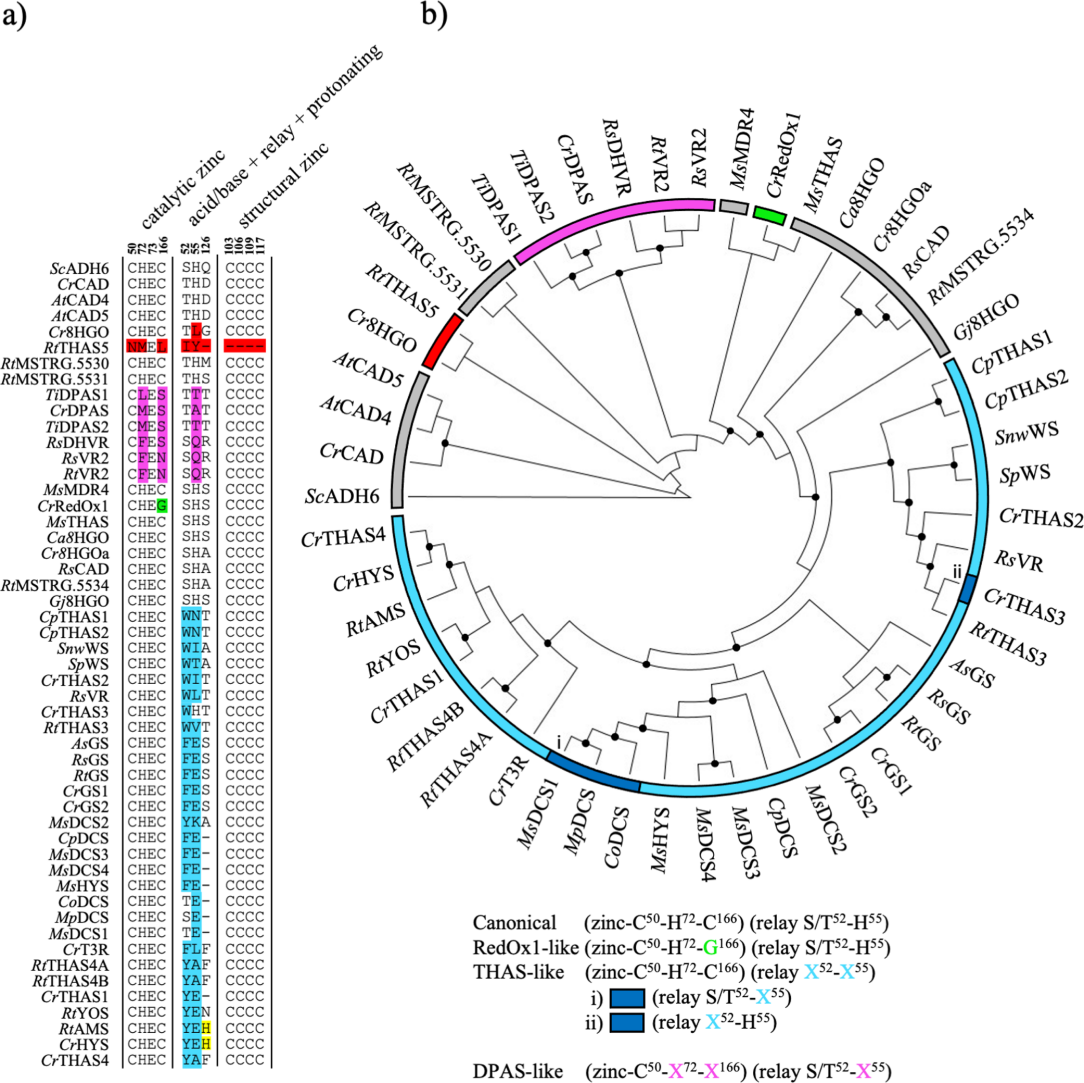
Sequence and phylogenetic analysis of characterized MIA MDRs. (a) Select positions from multiple sequence alignments of currently reported MIA MDRs (Table 1) and related CADs. Amino acid sequences were aligned using the G-INS-i strategy.^70^ Residues pertaining to zinc coordination and the proton relay are shown. The numbering based on *Cr*8HGOa is shown on top of the alignment. (b) Phylogenetic analysis of MIA MDRs and related CADs. The maximum-likelihood phylogenetic tree was constructed using IQ-tree with the LG + I + G4 substitution model^71,72^ from the multiple sequence alignment. The tree is rooted on ADH6 (KZV09178). Supported nodes are marked by black circles based on ultrafast bootstrap analysis using 1000 iterations (>95%)^73^ and branches were tested using SH-aLRT with 1000 replicates (>80%).^74^ Canonical catalytic architecture containing MDRs are highlighted in gray, THAS-like in cyan, DPAS-like in magenta, RedOx1-like in green, and MDRs not conforming to one of the described catalytic architectures in red. Their unique respective amino acid substitutions are highlighted in the multiple sequence alignment using the same coloring scheme.

### Sequential Iminium Reduction

Both DCS and DPAS can catalyze sequential reductions of unsaturated iminium species (Figures 3b and 4b).^28,39,41,42,45,63^ Initial 1,4-reduction produces an enamine intermediate that is presumably tautomerized to a 1,2-iminium species, which is then reduced in the second reduction. In the DCS catalyzed reduction of 4,21-dehydrogeissoschizine^45^ (Figure 3b) and the DPAS reduction of the late-stage biosynthetic intermediate 16­(*R*)-carbomethoxycleaviminium (Figure 4b),^63^ enamine tautomerization is likely facilitated by protonation of the enamine double bond. Additionally, DPAS can reduce precondylocarpine acetate twice to yield secodine (Figure 4b). In this case, enamine formation is facilitated by elimination of the acetoxyl group. Enamine tautomerization to the iminium could occur in the active site or, alternatively, the initial reduction product could tautomerize in solution and then rebind to the enzyme.

## Phylogenetic Analysis of Three Distinct Active Site Architectures

Discovery and characterization of these biosynthetic enzymes suggests that the active site of MDRs can catalyze 1,2-aldehyde, 1,2-iminium, 1,4-α,β-unsaturated iminium and 1,4-α,β-unsaturated aldehyde reductions. There appear to be three major classes of active site architectures that collectively, catalyze these reactions.^28^ The canonical architecture, which is responsible for oxidation of an alcohol to an aldehyde or the corresponding reduction of an aldehyde to an alcohol moiety, match the previously reported active sites of zinc-dependent MDRs.^23,24,26,27^ THAS-like MDRs catalyze the 1,2- and 1,4-reduction of iminium species. Typically, these enzymes have a modified catalytic zinc coordination sphere, and also lack the acid/base catalytic residue (serine/threonine) and proton relay. However, *Ms*DCS1, *Mp*DCS, and *Co*DCS, which produce (20*S*)-dihydrocorynantheine, retain the acid/base catalytic residue, and *Cr*THAS3, which produces the alkaloid tetrahydroalstonine, retains the proton relay. DPAS-like enzymes catalyze 1,2- and 1,4-reduction of iminium species and also catalyze 1,4-α,β-unsaturated aldehyde reduction. DPAS-like enzymes lack the catalytic zinc altogether but retain the acid/base catalytic serine/threonine. *Cr*RedOx1, which catalyzes a 1,2-iminium reduction, also lacks the catalytic zinc due to a H to G substitution in the coordination sphere.

Distinctive clades of THAS-like and DPAS-like MDRs are apparent in phylogenetic analyses of characterized MIA and CAD MDRs (Figure 7). Based on this phylogeny, the canonical MDRs are ancestral to THAS-like and likely also DPAS-like MDRs. Canonical zinc-dependent MDRs may have provided the ancestral architecture from which the new catalytic architectures in MIA MDRs evolved from. *Cr*8HGO did not clade close to other characterized canonical 8HGOs, including *Cr*8HGOa, congruent with its single substitution in the terminal proton relay histidine. Similarly, *Rt*THAS5, which catalyzes the 1,2-reduction of the iminium moiety of strictosidine aglycone to form tetrahydroalstonine, is also more distantly related. Multiple sequence alignments show that this enzyme lacks the canonical catalytic zinc coordinating or proton relay residues and is missing the loop that coordinates the structural zinc (Figure 7) and likely belongs to the quinone oxidoreductase family. *Ms*MDR4, *Ms*THAS, *Rt*MSTRG.5530, 5531, and 5534 also have canonical MDR catalytic architecture. *Ms*MDR4 and *Ms*THAS catalyze reductions of strictosidine aglycone and it is tempting to speculate that these enzymes represent an evolutionary bridge in the evolution of iminium reduction activity. *Ms*THAS catalyzes a 1,2-iminium reduction of strictosidine aglycone to form tetrahydroalstonine while *Ms*MDR4 was reported to convert strictosidine aglycone to an uncharacterized product.^46^

Collectively, these findings suggest that the noncanonical MIA MDR catalytic architectures in THAS-like, DPAS-like, and RedOx1-like clades evolved from zinc-dependent MDR ancestors that catalyze oxidation/reduction of alcohols/aldehydes. The noncanonical catalytic architectures in these MDRs may have arisen due to the more reactive nature of the iminium substrates. Reduction of strictosidine aglycone by a canonical MDR, possibly a CAD, may have provided the ancestral root of the current MIA MDRs. Indeed, strictosidine aglycone can be reduced by enzymes in both THAS-like and DPAS-like clades. Deeper phylogenetic and evolutionary investigation including ancestral reconstruction may help to elucidate the evolutionary history of the MIA ADHs.

### Recent Developments

During the preparation of this review several preprints were released reporting newly discovered MDRs in MIA biosynthesis. Briefly, two separate studies reported the discovery of MDRs catalyzing 1,2-iminium reductions involved in the unique stereochemical inversion of 3*S* to 3*R* MIAs in (3-dehydro-α-yohimbine reductases, DYR1 and DYR2)^75^ and heteroyohimbine/yohimbine/corynanthe C3-reductase (HYC3R) from , , (*Cr*THAS3), , , , , and .^61^ In phylogenetic analyses, HYC3Rs cladded with THAS-like MDRs and as indicative of the name, HYC3Rs demonstrate broad substrate range including strictosidine aglycone. Involvement of a non-MDR iminium reductase in 3*S* to 3*R* MIA inversion was also reported in .^76^ These recent developments showcase the relevance of these unique MDRs which have evolved to catalyze important reductions in MIA biosynthesis.

## Conclusions

MIA biosynthesis highlights how the enzymes of the MDR family can evolve to catalyze a broad range of reductive reactions. MDRs in this alkaloid pathway have evolved to catalyze 1,2 reductions of iminium species, 1,4 reductions of unsaturated iminium species, and 1,4-α,β-unsaturated aldehyde reductions. The inherent reactivity of iminium species has likely allowed disruptions to catalytic zinc binding, the proton relay or to the catalytic S/T residue. This range of chemical reactivity demonstrates the plasticity of the MDR active site. Phylogenetic analysis of the known MIA MDRs reveal three major catalytic architectures, canonical, THAS-like, and DPAS-like, which form well supported clades. This analysis may facilitate the identification and characterization of new MDR proteins.
